# Analysis of clinical presentations, lip transepidermal water loss and associated dermatological conditions in patients with chronic cheilitis

**DOI:** 10.1038/s41598-022-27115-9

**Published:** 2022-12-28

**Authors:** Yuanyuan Wang, Liqi Lin, Yuhong Wang, Minghui Wei, Jiao Wei, Yuan Cui, Yanfang Ren, Xinwen Wang

**Affiliations:** 1 Department of Oral Medicine, School of Stomatology, The Fourth Military Medical University (FMMU), Xi’an, China; 2 Shaanxi Clinical Research Center for Oral Diseases, The National Clinical Research Center for Oral Disease of China, Xi’an, China; 3 State Key Laboratory of Military Stomatology, Changle West Road 145, Xi’an, 710032 Shaanxi China; 4 Department of Periodontology and Oral Medicine, School of Stomatology, Lanzhou University, Lanzhou, China; 5 Eastman Institute for Oral Health, University of Rochester Medical Center, 625 Elmwood Ave., Rochester, NY 14620 USA

**Keywords:** Risk factors, Comorbidities

## Abstract

Chronic cheilitis (CC) is a spectrum of inflammatory changes of unknown etiology that affect the vermilion of the lips. This study aimed to describe the epidemiology, clinical presentations and risk factors of CC. Patients with CC were recruited from the National Clinical Research Center for Oral Disease of China. A convenience sample of inhabitants who live in the same geographical region were recruited as the control group. The lip skin transepidermal water loss (TEWL) and capacitance of CC patients were compared with that of age- and gender-matched controls. Our results demonstrated that of the 109 patients with CC, 72 (66.1%; 95% CI: 57.0–75.1%) were female. The common clinical presentations of CC consisted of desquamation (n = 99; 90.8%), and/or chapping (n = 81; 74.3%), and/or pruritus (n = 64; 58.7%). Multivariable analysis showed that allergic dermatologic diseases (*P* < 0.001; OR: 4.5; 95% CI: 2.4–8.4), anemia (*P* = 0.001; OR: 3.3; 95% CI: 1.5–7.5), and indoor/outdoor alternate working environment (*P* < 0.001; OR: 2.1; 95% CI: 1.5–2.8) were the significant risk factors for CC. The mean lip skin TEWL was found to be significantly higher, while the capacitance was lower in CC patients compared to that of control individuals. This study provides insights into the etiopathogenesis of CC and may help clinicians to identify the most effective management strategies.

## Introduction

Chronic cheilitis (CC) involves a spectrum of inflammatory changes of unknown etiology that affect the vermilion of the lips. CC has various manifestations and may present with desquamation, pruritus, chapping, or effusion. As a transitional zone between skin and mucosa, the vermilion of the lips comprises a thin stratum corneum that is devoid of both underlying salivary and sebaceous glands^1^. External etiological factors, such as windy and cold weather, lip licking or biting may promote dehydration of the lips, making the vermilion of the lips appear similar to CC. In addition, desquamation, pruritus or effusion may also appear as signs of other oral mucosal diseases such as oral lichen planus, orofacial granulomatosis, herpes, and oral candidiasis, which may lead to confusion in differential diagnosis CC from these conditions.

Due to the lack of a widely accepted diagnostic classification system, CC is often named after the characteristic signs and symptoms, for example, chapped lips, exfoliative cheilitis, cheilitis simplex, common cheilitis, cheilitis sicca, lip-licking cheilitis, angular cheilitis, irritant cheilitis, allergic contact cheilitis, and atopic cheilitis, etc^2–6^. The epidemiology, clinical presentations and skin barrier function of CC are poorly understood. In this study, we investigated the clinical characteristics of CC, defined the risk factors associated with CC, and assessed the skin barrier function of CC patients using a case–control study design that allowed exclusion of some confounding factors.

## Methods

### Study population

We recruited 109 patients with CC via the Department of Oral Medicine between December 2018 and June 2019. Migration to different geographic locations may lead to changes in the environment (including humidity, ultraviolet intensity, temperature, etc.), which might affect the risk and severity of CC. Therefore, the survey was conducted among residents who lived in Xi’an city (Longitude: 108, latitude: 34), located in central China, with an average temperature of 14.7 °C (± 14.65 °C), approximately 90 sunny days per year and semi-humid climate.

The diagnosis of CC was based on the history and clinical findings of crusting, scaling, peeling or chapping of one or both lips that had been present for at least 8 weeks, excluding the cases related to mechanical stimuli. As several variants of CC may be present at different time in the same patient, we made a unified diagnosis of chronic cheilitis, no longer distinguished chapped lips, exfoliative cheilitis, cheilitis simplex, common cheilitis, cheilitis sicca, allergic contact cheilitis, and atopic cheilitis. A biopsy is necessary for further histologic assessment to exclude granulomatous cheilitis, cheilitis glandularis, actinic cheilitis and other associated diseases, details of the diagnostic criteria used are described in Table 1.

**Table 1:** Diagnostic investigations and criteria of chronic cheilitis.

Investigations	Results
Occlusion of the mouth	Should be normal
Dentures	Should be fitting
Oral ulcers/erosion/streaks/patch/ nodular /pseudomembrane	Should be negative
Lip licking/thumb sucking/lip biting/ history of surgery or trauma	Excluded
**Histopathology**	
Granulomas, fibrosis, dense subepithelial infiltration of lymphocytes, liquefaction degeneration of basal epithelial cells, peculiar keratotic plug, Russel’s body, dysplasia in epidermis, inflammatory cells around salivary glands/ blood vessels, lymphoid follicle-like structure	Should be negative^a^

^a^Biopsy was not obtained as part of the diagnostic work-up, but was necessary when there are clinical features compatible with potential diagnosis of other mucosa diseases, such as granulomatous cheilitis, actinic cheilitis, Melkersson–Rosenthal syndrome, cheilitis glandularis, oral lichen planus, discoid lupus erythematosus, etc.

Control individuals were a convenience sample of community-dwelling individuals, who live in the same geographical region, or other patients visiting the Stomatological Hospital of the Fourth Military Medical University (FMMU) for routine dental exams (n = 208). Proportional gender and age matching were used to select controls for the measurement of skin barrier function, and the controls must not have signs and symptoms of CC.

### Variables

Details of the demographics and medical history were collected through questionnaire. The medical histories of their skin problems and previous dermatological diagnoses were further evaluated by qualified dermatologists. Hematological data within one year were collected in participants as they were required: (1) for medical diagnostic purposes; (2) for routine examination; (3) to monitor therapeutic outcomes. For those with a confirmed diagnosis of CC, the data, including the duration of CC, presented symptoms, involved sites, and aggravating factors were recorded.

### Measurements

Passive diffusion of water through the stratum corneum, also known as transepidermal water loss (TEWL), and capacitance, which reflects water content of stratum corneum are widely applied for evaluating the skin barrier function^7,8^. Measurement of TEWL and capacitance allows objective assessment of the functional status of the skin barrier^9,10^. In this study TEWL was measured with TewameterTM300 (Courage Khazaka, Germany). Capacitance was measured using the CorneometerCM825 (Courage Khazaka, Germany).

Lip measurements were performed on the lower lip vermilion, while the skin measurements were performed on the forearm. The participants applied no topical agents on their lips and skin for at least 12 h. Before the measurement, the lips of the patients were wiped using paper towels and acclimated for 30 min in the testing room. All measurements were conducted in an environment with a temperature of 22–27 °C and relative humidity of 45–50%. Control data of TEWL and capacitance were obtained from the control population by matching the patients at a ratio of 1:1 based on age and sex.

### Statistical analysis

The data obtained were recorded and processed using SAS 9.4 software (SAS Institute, Cary, NC, USA). Categorical and continuous variables were compared using χ^2^, Fisher's exact, and t-tests, respectively. Logistic regression modeling was used to evaluate the risk factors. The interaction terms for statistically significant effect modifiers were added to the multivariate model while calculating appropriate odds ratio (OR) and 95% confidence intervals (CI). Two-sided P < 0.05 was considered statistically significant.

### Ethics approval

The study protocol was reviewed and approved by the Ethics Committee of School of Stomatology, the Fourth Military Medical University (IRB number: IRB-REV-2020041). The study was carried out in accordance with the ethical standards of Helsinki Declaration and its amendments or comparable ethical standards.

### Informed consent

Before starting the study, the participants were informed about the study and signed informed consent forms. The participants and any identifiable individuals consented to publication of his/her image.

## Results

### Demographic details of CC patients

Our study included 109 CC patients, whose median age (and range) was 27 years (5–59 years). Of the 109, 72 (66.1%; 95% CI: 57.0–75.1%) were female, with a peak in the incidence rate seen during the third and fourth decade of their life. The median age (and range) for females was 29 years (5–59 years), and for males was 26 years (8–56 years). The duration of symptoms before definitive diagnosis was over 1 year for the majority of CC patients (76.1%; 95% CI: 68.0–84.3%).

### Clinical presentations of CC

**Figure 1:**
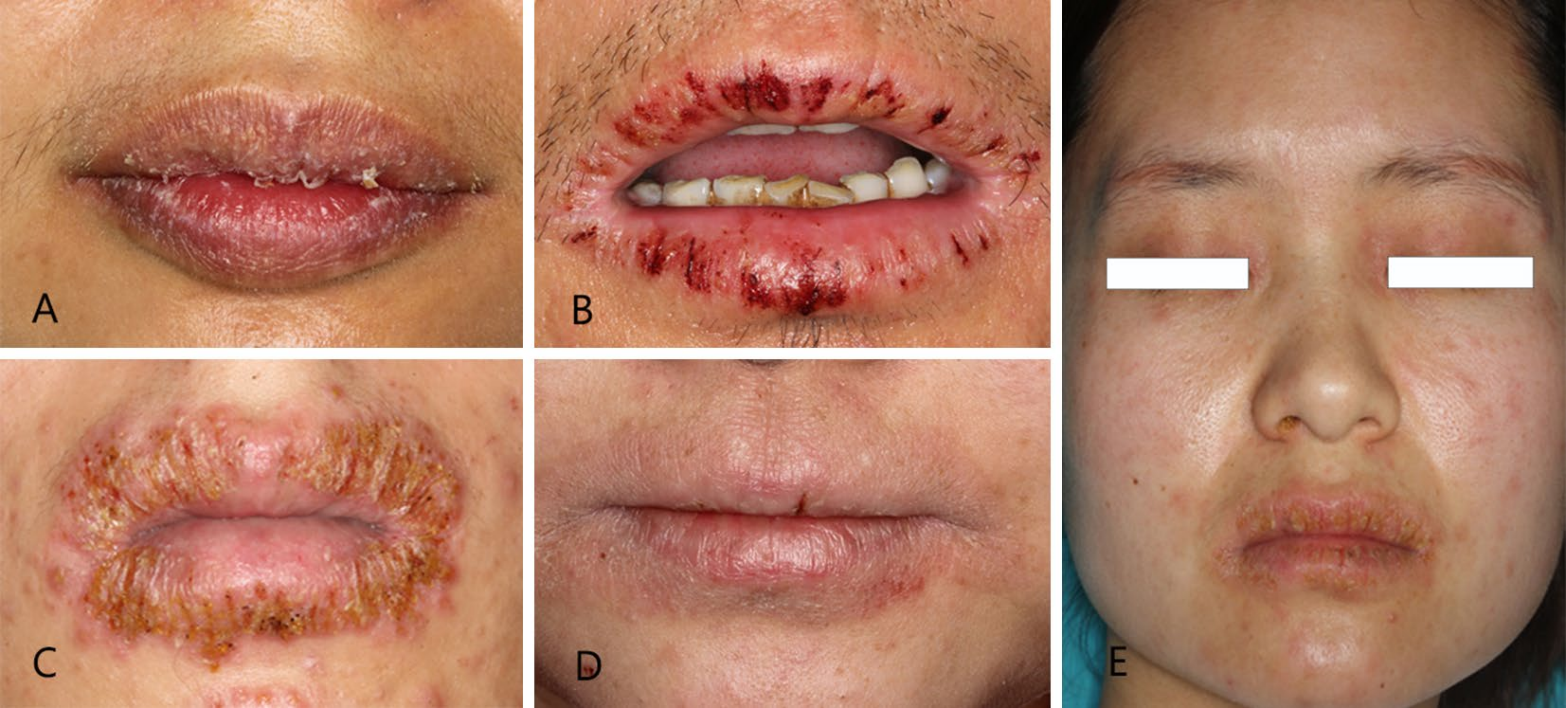
Phenotypes of chronic cheilitis. (**A**) Desquamation, (**B**) chapping, (**C**) effusion/eschar, (**D**) lack of color in the lip, (**E**) severe chronic cheilitis in a female patient with extensive lesions on the paraorbital, perioral and cheek skin.

Both lips were affected in 103 (94.5%; 95% CI: 90.1–98.8%) patients. Five patients had CC on the lower lip only and one patient on the upper lip only. CC displays a spectrum of clinical manifestations (Fig. 1), common clinical presentations consisted of desquamation (n = 99; 90.8%) and/or chapping (n = 81; 74.3%) and/or pruritus (n = 64; 58.7%). Additional details about the range and frequency of the presented symptoms are provided in Table 2.

**Table 2:** Clinical features of 109 patients with chronic cheilitis.

Features	No. (%)
**Duration**	
Within 1 year	26 (23.85)
Over 1 year	83 (76.15)
**Presenting symptoms**	
Desquamation	99 (90.83)
Chapping	81 (74.31)
Pruritus	64 (58.72)
Effusion/escharosis	45 (41.28)
Papule	41 (37.61)
Others	6 (5.50)
**Involved sites**	
Upper lip	1 (0.92)
Lower lip	5 (4.59)
Both lips	103 (94.50)
**Aggravating factors**	
Dryness	72 (66.06)
Spicy food	58 (53.21)
Seasonal variation	54 (49.54)
Cosmetics	42 (38.53)
Cold environment	34 (31.19)
Sunlight	24 (22.02)
Hot environment	20 (18.35)
Stress	10 (9.17)
Others	20 (18.35)

For the nine surveyed factors, dryness was the most frequent aggravating factor (66.1%), followed by spicy food (53.2%), seasonal variation (49.5%), cosmetics (38.5%), and cold environment (31.2%) (Table 2).

### Risk factors for CC

In the univariate analysis, gender, occupation, skin disease, and anemia showed odds ratios (ORs) with a significance of P < 0.05, which were entered into the multivariable analysis (Table 3). The final multivariable model demonstrated that skin disease (P < 0.001; OR: 4.5; 95% CI: 2.4–8.4), anemia (P = 0.001; OR: 3.3; 95% CI: 1.5–7.5), and indoor/outdoor alternate working environment (P < 0.001; OR: 2.1; 95% CI: 1.5–2.8) were the significant risk factors for CC (Table 3). Of the 20 CC patients with anemia, 19 (95.0%; 95% CI: 84.5–105.5%) had iron deficiency anemia and one (5.0%; 95% CI: 0–15.5%) had megaloblastic anemia. Among the total 39 CC patients with skin diseases, 16 (41.0%; 95% CI: 24.9–57.2%) had a history of urticaria, 14 (35.9%; 95% CI: 20.1–51.7%) had atopic dermatitis (AD) or eczema, and 9 patients (23.1%; 95% CI: 9.2–36.9%) had allergic contact dermatitis (ACD).

**Table 3:** Risk factors associated with chronic cheilitis.

Variable	No. (%)		Unadjusted OR (95% CI)	Adjusted OR (95% CI)
	CC (N = 109)	Control (N = 208)		
Age, years	27 (± 10)	28 (± 9)	1.02 (0.96, 1.01)	–
**Gender**				
Female	72 (66.06)	112 (53.85)	1.67 (1.03, 2.70)*	1.37 (0.80, 2.34)
Male	37 (33.94)	96 (46.15)		
**Occupation**				
Indoor	63 (57.80)	170 (81.73)	–	–
Outdoor	4 (3.67)	7 (3.37)	1.54 (0.44, 5.45)	–
Indoor/outdoor	42 (38.53)	31 (14.90)	3.66 (2.12, 6.32)***	2.08(1.54, 2.81)***
**Education level (completed years of schooling)**				
10–16	90 (82.57)	186 (89.42)	–	–
≤ 9	18(16.51)	19(9.13)	0.44 (0.17, 1.10)	–
> 16	1(0.92)	3(1.44)	0.70 (0.26, 1.86)	–
Rhinitis	33 (30.28)	80 (38.46)	0.70 (0.42, 1.14)	–
Asthma	2 (1.83)	7 (3.37)	0.54 (0.11, 2.63)	–
Skin diseases	39 (35.78)	25 (12.02)	4.08 (2.30, 7.23)***	4.54(2.45, 8.42)***
Anemia	20 (18.35)	13 (6.25)	3.37 (1.61, 7.08)**	3.33(1.48, 7.52)**
**Family history**				
Rhinitis	30 (27.52)	54 (25.96)	1.08 (0.64, 1.83)	–
Asthma	4 (3.67)	7 (3.37)	1.09 (0.31, 3.82)	–
Skin diseases	10 (9.17)	12 (5.77)	1.65 (0.69, 3.95)	–

*CC* chronic cheilitis, *OR* odds ratio, *CI* confidence interval.

*P < 0.05, **P < 0.01, ***P < 0.001.

### The skin barrier function of CC patients

**Figure 2:**
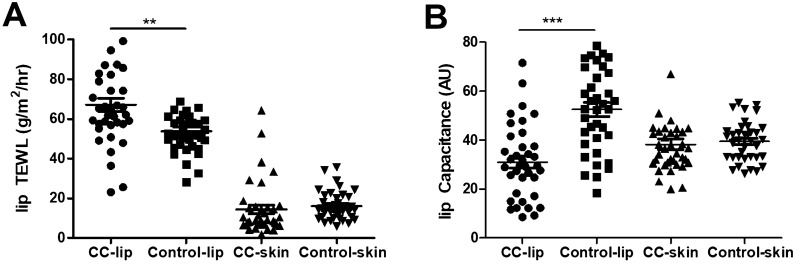
The biophysical parameter values in chronic cheilitis patients. (**A**) TEWL values in the lip and forearm of CC patients and control individuals, (**B**) capacitance values in the lip and forearm of CC patients and control individuals. *CC* chronic cheilitis, *TEWL* transepidermal water loss; **P < 0.01, ***P < 0.001.

Thirty-seven CC patients and 37 control individuals (23 female, 14 male, 28 ± 7 years vs. 27 ± 7 years; P > 0.05) had participated in the biophysical measurements in the lip and forearm skin. The TEWL and capacitance values of lip in the CC group were distributed with a mean of 66.8 g/m^2^·h (± 20.9 g/m^2^·h) and 31.0 AU (± 15.2 AU). Compared to the TEWL and capacitance values of the lip in the age and gender-matched control group, the TEWL value of the lip was significantly higher (P < 0.01) in the CC group, while the capacitance value was found to be significantly lower (P < 0.001) (Fig. 2). The TEWL and capacitance values of arm skin showed no significant differences between the two groups.

## Discussion

The data from our study of 109 CC patients showed that CC primarily affected young patients (mean age of 27 years), wherein two-thirds were female, with 94.5% of them showing involvement of both lips. Desquamation, chapping, and pruritus were the most frequently presented symptoms, however the chronic course of CC was more likely to be dynamically diverse, with the severity and distribution of CC lesions influenced by environmental factors, particularly dryness, food, and cosmetics.

Dryness was shown to be an important aggravating factor for CC in the study, which was also reflected in the influence of seasonal variation and occupation. Table 3 shows that the occupation that involved frequent indoor/outdoor environmental transition was significantly related to CC. CC patients emphasized that moving from outside to a warm room during winter had an aggravating effect on CC, which may attribute to the dramatically reduced humidity in air upon heating^11^. It is noted, our study showed that the population with a history of allergic dermatologic diseases including urticaria, AD and ACD, was at increased risk for CC occurrence. Likewise, previous studies demonstrated that dryness was correlated well with the severity of AD and ACD^12,13^ as a result of significant decrease in the skin barrier function^14^, which further highlight the close relationship between CC and allergic dermatologic disease. In agreement with this, a recent cross-sectional study which examined the multiple factors involved in the aetiology of CC showed that of the systemic diseases recorded among CC patients, skin diseases were noticed most frequently although the type of skin disease was not described in detail^15^. Another study, which investigated the prevalence of hypersensitivity in cheilitis patients demonstrated increased serum total IgE level and positive food-specific IgG in cheilitis patients compared to the healthy control^16^.

Previously, atopic cheilitis manifesting as exfoliative cheilitis was described in cases of AD^17^, some cheilitis cases (known as allergic cheilitis) were regarded as an allergic contact eczema of the lips in reaction to an exogenous substance^5,18^. However, the association between CC and dermatologic disease has not yet been validated in CC patients. In the current case–control study, allergic dermatologic disease (OR: 4.5; 95% CI: 2.4–8.4) was confirmed as a potential risk factor for occurrence of CC. We found that urticaria was the most frequent (41.0%) dermatologic disease associated with CC, followed by AD/eczema (35.9%) and ACD (23.1%).

AD, urticaria, and ACD represent three important allergic dermatologic diseases, the pathogenesis of them is not completely understood. Studies emphasized Th2 cytokines as the primary stimuli for the “atopy” of AD^19^, and that ACD is a type IV cutaneous cell-mediated hypersensitivity reaction triggered by environmental allergens^20^, whereas urticaria is an IgE-mediated allergic dermatologic disease^21^. However, their pathophysiology is likely multifactorial, and an intricate relationship exists among these diseases, which involves immune, skin barrier and environmental factors^22–24^. ACD is often included in the differential diagnosis for AD as they can be similar in their clinical presentations, and AD was considered an important risk factor for the occurrence of urticaria^25^. Among inflammatory mediators of urticaria, some are also implicated in the pathogenesis of AD^26^.

Skin barrier dysfunction plays a crucial role in the pathogenesis of AD^27^. Moreover, studies have shown that an increased skin permeability induced by an impaired skin barrier could provide an opportunity to effectively access the allergens in the viable epidermis. This evokes innate signaling pathways during the sensitization phase, which is required for the activation of the immune response. Thus, skin barrier dysfunction enhances the acquisition of allergic dermatologic diseases^28–32^. We measured TEWL and capacitance in the lower lip and forearm skin, and found that compared to the TEWL and capacitance of lip in the control group, TEWL in the CC group was significantly higher while the capacitance was significantly lower, indicating an impaired skin barrier function in the lip of the CC patients. These findings were consistent with the observations made in the skin lesions of AD and ACD patients^33,34^. However, the TEWL and capacitance of arm skin showed no significant differences between the two groups. Hence, the possibility that the defective skin barrier function of the lip is mediated by the inflammatory reaction cannot be excluded^35^. The mechanism associated with this phenomenon warrants further investigation.

The association between atopic disorders (ie, asthma, eczema and food allergy) and anemia was reported previously. From a survey that included 207,007 children and adolescents in US, atopic disorders were shown to be associated with increased odds of anemia, and the odds of anemia increased with the number of atopic disorders present^36^. Nevertheless, there is a paucity of data explaining such association. A recent study using animal model demonstrated anemia was associated with decreased epithelial barrier function due to down-regulation of tight junction proteins^37^, which is in agreement with the survey and our study. But our findings still indicate a need for careful evaluation of the implications of anemia in CC.

In summary, use of case definition to improve diagnostic specificity yielded consistent results, allowing us to study the risk factors for CC. To our knowledge, this is the first study to examine the skin barrier function of CC patients, which increases our understating of the disease. This study also has limitations. The positive association of CC with allergic dermatologic diseases was confirmed in the case–control study, however patch test, biopsy or serum cytokine test were not performed for most of the patients, and more detailed data were not collected, which might demonstrate additional systematic risk factors linked to CC.

## Conclusion

The morbidity of CC is closely associated with allergic dermatologic diseases, and the lip lesion of CC is characterized by impaired skin barrier function. The study provides insight into the etiopathogenesis of CC and may help clinicians to identify the most effective management strategies.

## Data Availability

The datasets generated and/or analyzed during the current study are available from the corresponding author on reasonable request.
